# Supplementary material for: Gender versus sex in predicting outcomes of traumatic brain injury: a cohort study utilizing large administrative databases
Source: Sci Rep. 2023 Oct 27;13:18453. doi: 10.1038/s41598-023-45683-2 (PMC10611793; doi:10.1038/s41598-023-45683-2)
Supplement: Supplementary file 1 — Supplementary Information. [file 41598_2023_45683_MOESM1_ESM.docx]

**Title:** Gender versus sex in predicting outcomes of traumatic brain injury: A cohort study utilizing large administrative databases

Supplementary Information

**Author list and affiliations:** Anastasia Teterina, PhD^1^, Suvd Zulbayar, MSc^1^, Tatyana Mollayeva, MD, PhD ^1-4^, Vincy Chan, MPH, PhD^2-5^, Angela Colantonio, PhD ^1-5^, Michael Escobar, PhD^1^

^1^ Dalla Lana School of Public Health, University of Toronto, Canada

^2^ KITE-Toronto Rehabilitation Institute, University Health Network, Canada

^3^ Rehabilitation Sciences Institute, Temerty Faculty of Medicine, University of Toronto, Canada

^4^ Acquired Brain Injury Research Lab, University of Toronto, Canada

^5^ Institute of Health Policy, Management and Evaluation, University of Toronto, Canada

**Supplementary Figure 1. Gender score distribution in males and females within age groups, test dataset (N=68,900).**


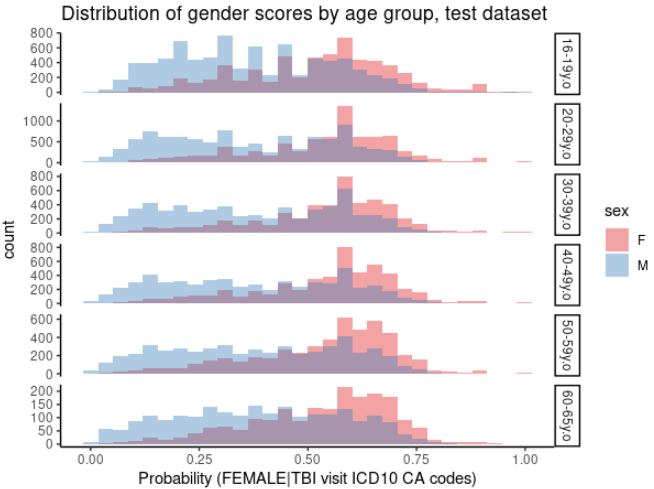


**Supplementary Table 1. Severity distribution for training set (N=85,889) by mortality group.** Data given in n (%).

Abbreviations: TBI = Traumatic Brain Injury.

*Survival status at day 30 from the first TBI event

| **Parameter** | **Died within 30 days*** | **Alive after 30 days*** |
| --- | --- | --- |
|  | **N=1,351** | **N=84,538** |
| *TBI severity* |  |  |
| Unknown | 264 (19.5) | 47,332 (56.0) |
| Mild | 123 (9.1) | 25,856 (30.6) |
| Moderate | 97 (7.2) | 3,389 (4.0) |
| Severe | 867 (64.2) | 7,961 (9.4) |

**Supplementary Table 2. ADG score distribution for training set (N=85,889) by mortality group.** Data given in n (%). To prevent breach of confidentiality for ICES data, small categories are combined for reporting and percentages are reported without absolute numbers.

Abbreviations: ADG = Johns Hopkins’ Aggregated Diagnosis Groups

*Survival status at day 30 from the first TBI event

| **Parameter** | **Died within 30 days*** | **Alive after 30 days*** |
| --- | --- | --- |
|  | **N=1,351** | **N=84,538** |
| *ADG score* |  |  |
| 0 | 10 (4.42) | 216 (95.58) |
| 1 | 276 (1.09) | 25,023 (98.91) |
| 2 | 328 (1.22) | 26,455 (98.78) |
| 3 | 225 (1.67) | 13,281 (98.33) |
| 4 | 137 (1.78) | 7,558 (98.22) |
| 5 | 94 (2.08) | 4,427 (97.92) |
| 6 | 64 (2.29) | 2,278 (97.71) |
| 7 | 56 (3.12) | 1,738 (96.88) |
| 8 | 39 (3.67) | 1,025 (96.33) |
| 9 | 25 (3.21) | 753 (96.79) |
| 10 | 22 (4.42) | 476 (95.58) |
| 11 | 29 (8.90) | 297 (91.10) |
| 12 | 15 (6.79) | 206 (93.21) |
| 13 | 11 (7.43) | 137 (92.57) |
| 14 | 10 (9.71) | 93 (90.29) |
| 16-21 | 10 (7.58) | 122 (92.42) |

**Supplementary Table 3. Characteristics of discharge location analysis by group.** Data given as median (Q1, Q3) for continuous variables or (%) for categorical variables.

Abbreviations: Q1 = 1^st^ quartile; Q3 = 3^rd^ quartile; LTC = Long Term Care; CCC = Inpatient Complex Continuing Care; ADG = Johns Hopkins’ Aggregated Diagnosis Groups.

*Other contains the categories: transfer to another inpatient care facility, addiction treatment centers, signing out against medical advice.

| **Parameter** | **Home** | **Home with support** | **LTC & CCC** | **Other*** | **Rehab** |
| --- | --- | --- | --- | --- | --- |
|  | **N=4,427** | **N=645** | **N=155** | **N=1,196** | **N=920** |
| *Sex (%)* |  |  |  |  |  |
| Female | 26.8 | 31.5 | 32.3 | 25.5 | 27.9 |
| *Age (years)* |  |  |  |  |  |
| Median | 40 | 48 | 50 | 45 | 42 |
| Q1 | 24 | 33 | 40 | 29.5 | 26 |
| Q3 | 53 | 58 | 59 | 56 | 55 |
| *Rural (%)* |  |  |  |  |  |
| Yes | 16.4 | 15.7 | 14.2 | 20.1 | 12.6 |
| *Income quintile (%)* |  |  |  |  |  |
| 1 (lowest) | 24.0 | 26.1 | 27.1 | 26.8 | 24.2 |
| 2 | 20.1 | 18.3 | 26.5 | 20.4 | 22.3 |
| 3 | 18.1 | 19.8 | 15.5 | 19.5 | 19.1 |
| 4 | 19.5 | 19.4 | 18.1 | 17.2 | 18.5 |
| 5 (highest) | 18.3 | 16.4 | 12.9 | 16.1 | 15.9 |
| *Length of Stay (days)* |  |  |  |  |  |
| Median | 3 | 9 | 23 | 8 | 23 |
| Q1 | 1 | 5 | 11 | 2 | 12 |
| Q3 | 5 | 16 | 54 | 17 | 41 |
| *ADG score* |  |  |  |  |  |
| Median | 2 | 3 | 3 | 3 | 2 |
| Q1 | 1 | 2 | 1 | 2 | 1 |
| Q3 | 3 | 4 | 6 | 5 | 3 |
| *Gender score* |  |  |  |  |  |
| Median | 0.27 | 0.28 | 0.29 | 0.24 | 0.23 |
| Q1 | 0.15 | 0.14 | 0.14 | 0.12 | 0.12 |
| Q3 | 0.41 | 0.41 | 0.42 | 0.36 | 0.36 |

**Supplementary Table 4. Characteristics of TBI-related mortality analysis by group.** Data given as median (Q1, Q3) for continuous variables or (%) for categorical variables. To prevent breach of confidentiality for ICES data, small categories are combined for reporting and percentages are reported without absolute numbers.

Abbreviations: Q1 = 1^st^ quartile; Q3 = 3^rd^ quartile; ADG = Johns Hopkins’ Aggregated Diagnosis Groups; MVC = Motor-Vehicle Collisions.

*One subject can have several causes

**From the first TBI event

| **Parameter** | **Died within 30 days**** | **Alive after 30 days**** |
| --- | --- | --- |
|  | **N=402** | **N=3,987** |
| *Sex (%)* |  |  |
| Female | 29.9 | 28.9 |
| *Age (years)* |  |  |
| Median | 48.5 | 44 |
| Q1 | 34 | 28 |
| Q3 | 57 | 56 |
| *Rural (%)* |  |  |
| Yes | 13.7 | 12.5 |
| *Income quintile (%)* |  |  |
| 1 (lowest) | 26.6 | 23.0 |
| 2 | 23.1 | 20.1 |
| 3 | 19.2 | 18.9 |
| 4 | 16.4 | 19.2 |
| 5 (highest) | 14.7 | 18.1 |
| *ADG score* |  |  |
| Median | 3 | 2 |
| Q1 | 2 | 1 |
| Q3 | 5 | 4 |
| *Gender score* |  |  |
| Median | 0.31 | 0.28 |
| Q1 | 0.19 | 0.15 |
| Q3 | 0.4 | 0.4 |
| *Cause of injury* (%)* |  |  |
| Falls | 44.3 | 42.1 |
| Struck by or Cyclist Collision | 3.2 | 19.3 |
| MVC | 33.6 | 23.2 |
| Other | 20.2 | 16.3 |

**Supplementary Table 5. ICD-10-CA diagnostic codes with higher odds of being woman-like.** Ranked in a descending order according to their validation OR.

Abbreviations: ICD-10-CA = International Statistical Classification of Diseases and Related Health Problems, Tenth Revision, Canada, OR = odds ratio, CI = confidence interval.

| **ICD-10-CA Code** | **Description** | **Training** | **Validation** |
| --- | --- | --- | --- |
|  |  | **OR (95% CI)** | **OR (95% CI)** |
| T741 | Physical abuse | 13.12 (4.71, 36.58) | 27.16 (3.75, 196.62) |
| Y070 | By spouse or partner | 41.54 (19.61, 88.01) | 18.11 (8.86, 37.01) |
| W04 | Fall while being carried or supported by other persons | 7.57 (4.72, 12.15) | 10.95 (5.01, 23.92) |
| U99064 | Aesthetic sports | 27.47 (3.79, 198.91) | 9.88 (2.29, 42.64) |
| V800 | Animal-rider or occupant of animal-drawn vehicle injured by fall from or being thrown from animal or animal-drawn vehicle in noncollision accident | 9.09 (7.6, 10.86) | 8.81 (6.85, 11.35) |
| U99068 | Other specified gymnastic and aesthetic sports and recreational activity | 7.5 (2.91, 19.31) | 7.41 (2.18, 25.14) |
| Z630 | Problems in relationship with spouse or partner | 3.64 (1.89, 7.02) | 7.41 (2.18, 25.14) |
| W54 | Bitten or struck by dog | 5.43 (3.76, 7.85) | 6.47 (3.57, 11.71) |
| U99037 | Horse riding sports | 10.31 (5.17, 20.57) | 6.18 (2.89, 13.2) |
| V809 | Animal-rider or occupant of animal-drawn vehicle injured in other and unspecified transport accidents | 4.22 (2.41, 7.36) | 4.73 (1.93, 11.62) |
| W1800 | Fall on same level in or from bathtub | 3.06 (2.28, 4.1) | 4.1 (2.63, 6.42) |
| Y05 | Sexual assault by bodily force | 3.12 (1.68, 5.8) | 3.86 (1.74, 8.55) |
| W55 | Bitten or struck by other mammals | 3.02 (2.49, 3.67) | 3.43 (2.6, 4.52) |
| G439 | Migraine, unspecified | 3.07 (2.4, 3.92) | 3.21 (2.25, 4.58) |
| U99042 | Curling | 2.21 (1.41, 3.45) | 2.78 (1.41, 5.48) |
| W08 | Fall involving other furniture | 3.16 (2.35, 4.25) | 2.75 (1.85, 4.07) |
| V484 | Car occupant injured in noncollision transport accident, while boarding or alighting | 2.16 (1.64, 2.86) | 2.72 (1.77, 4.19) |
| W1801 | Fall on same level in or from shower stall | 2.97 (2.22, 3.98) | 2.55 (1.68, 3.86) |
| S060 | Concussion | 2.42 (2.37, 2.47) | 2.44 (2.36, 2.52) |
| V495 | Passenger of car injured in collision with other and unspecified motor vehicles in traffic accident | 2.16 (1.67, 2.79) | 2.43 (1.61, 3.68) |
| U99001 | Volleyball | 2.38 (1.65, 3.45) | 2.31 (1.39, 3.83) |
| R111 | Nausea alone | 2.02 (1.47, 2.77) | 2.28 (1.49, 3.49) |
| S1340 | Whiplash associated disorder [WAD1] with complaint of neck pain, stiffness or tenderness only | 2.44 (2.15, 2.76) | 2.28 (1.91, 2.71) |
| W0202 | Fall involving roller skates/in-line skates | 2.89 (2.12, 3.93) | 2.26 (1.5, 3.41) |
| V436 | Car occupant injured in collision with car, pick-up truck or van, passenger, traffic accident | 2.36 (2.15, 2.59) | 2.25 (1.98, 2.56) |
| U9828 | Place of occurrence, school and other institutions and.public areas | 2.07 (1.85, 2.31) | 2.21 (1.9, 2.58) |
| W07 | Fall involving chair | 2.48 (2.11, 2.93) | 2.19 (1.74, 2.76) |
| V439 | Car occupant injured in collision with car, pick-up truck or van, unspecified car occupant, traffic accident | 1.92 (1.58, 2.34) | 2.15 (1.54, 3.01) |
| W5108 | Striking against or bumped into by another person in non-sports | 2.34 (1.95, 2.81) | 2.04 (1.59, 2.61) |
| U99041 | Ice skating | 2.77 (2.02, 3.8) | 2.02 (1.31, 3.12) |
| W00 | Fall on same level involving ice and snow | 1.97 (1.87, 2.07) | 2 (1.86, 2.16) |
| W01 | Fall on same level from slipping, tripping and stumbling | 2.06 (1.97, 2.16) | 2 (1.88, 2.12) |
| W06 | Fall involving bed | 2.11 (1.76, 2.52) | 1.81 (1.42, 2.3) |
| W2208 | Striking against or struck by/in non-sports | 1.68 (1.62, 1.74) | 1.71 (1.62, 1.79) |
| S136 | Sprain and strain of joints and ligaments of other and unspecified parts of neck | 2.09 (1.9, 2.31) | 1.68 (1.47, 1.91) |
| W2100 | Striking against or struck by ball | 1.65 (1.54, 1.77) | 1.65 (1.5, 1.82) |
| S300 | Contusion of lower back and pelvis | 1.59 (1.21, 2.09) | 1.64 (1.16, 2.32) |
| V892 | Person injured in unspecified traffic motor-vehicle accident | 1.45 (1.27, 1.65) | 1.62 (1.36, 1.94) |
| W1809 | Other and unspecified fall on same level | 1.57 (1.45, 1.7) | 1.62 (1.44, 1.82) |
| W10 | Fall on and from stairs and steps | 1.51 (1.44, 1.59) | 1.58 (1.47, 1.69) |
| U980 | Place of occurrence, home | 1.47 (1.42, 1.53) | 1.49 (1.42, 1.57) |
| W2209 | Striking against or struck by unspecified | 1.61 (1.47, 1.76) | 1.47 (1.29, 1.67) |
| V435 | Car occupant injured in collision with car, pick-up truck or van, driver, traffic accident | 1.36 (1.29, 1.44) | 1.45 (1.35, 1.56) |
| S1348 | Other sprain and strain of cervical spine | 1.88 (1.63, 2.17) | 1.41 (1.16, 1.71) |
| R51 | Headache | 1.24 (1.14, 1.34) | 1.4 (1.25, 1.58) |
| W19 | Unspecified fall | 1.4 (1.34, 1.47) | 1.38 (1.29, 1.48) |
| R55 | Syncope and collapse | 1.5 (1.37, 1.65) | 1.38 (1.21, 1.58) |
| W18 | Other fall on same level | 1.41 (1.32, 1.52) | 1.34 (1.21, 1.48) |
| W0200 | Fall involving ice skates | 1.37 (1.24, 1.51) | 1.27 (1.1, 1.46) |
| U989 | Unspecified place of occurrence | 1.17 (1.15, 1.2) | 1.17 (1.14, 1.21) |
| S06000 | Concussion without loss of consciousness without open intracranial wound | 0.93 (0.9, 0.96) | 0.92 (0.88, 0.97) |
| U984 | Place of occurrence, street and highway | 0.86 (0.81, 0.91) | 0.85 (0.79, 0.92) |
| U985 | Place of occurrence, trade and service area | 0.85 (0.81, 0.9) | 0.84 (0.78, 0.91) |
| X599 | Exposure to unspecified factor causing other and unspecified injury | 0.85 (0.78, 0.91) | 0.81 (0.73, 0.9) |
| V475 | Car occupant injured in collision with fixed or stationary object, driver, traffic accident | 0.79 (0.71, 0.89) | 0.76 (0.64, 0.9) |
| W0204 | Fall involving snowboard | 0.73 (0.66, 0.81) | 0.75 (0.65, 0.87) |
| S06900 | Intracranial injury, unspecified without loss of consciousness without open intracranial wound | 0.77 (0.67, 0.88) | 0.74 (0.61, 0.9) |
| W17 | Other fall from one level to another | 0.66 (0.6, 0.73) | 0.73 (0.64, 0.83) |
| S06800 | Other intracranial injuries without loss of consciousness without open intracranial wound | 0.73 (0.63, 0.85) | 0.7 (0.57, 0.87) |
| U981 | Place of occurrence, residential institution | 0.68 (0.6, 0.76) | 0.64 (0.54, 0.75) |
| S007 | Multiple superficial injuries of head | 0.6 (0.49, 0.74) | 0.63 (0.48, 0.83) |
| U983 | Place of occurrence, sports and athletics area | 0.63 (0.61, 0.66) | 0.62 (0.59, 0.66) |
| W5104 | Striking against or bumped into by another person in soccer | 0.61 (0.54, 0.69) | 0.61 (0.52, 0.73) |
| W5103 | Striking against or bumped into by another person in football/rugby | 0.52 (0.47, 0.57) | 0.61 (0.54, 0.69) |
| I100 | Benign hypertension | 0.6 (0.54, 0.67) | 0.6 (0.52, 0.69) |
| S32400 | Fracture of acetabulum, closed | 0.6 (0.49, 0.73) | 0.59 (0.45, 0.79) |
| S06600 | Traumatic subarachnoid haemorrhage without loss of consciousness without open intracranial wound | 0.53 (0.44, 0.64) | 0.58 (0.45, 0.75) |
| Z721 | Alcohol use | 0.56 (0.46, 0.68) | 0.58 (0.44, 0.76) |
| W2203 | Striking against or struck by/playing football/rugby | 0.63 (0.53, 0.76) | 0.58 (0.44, 0.75) |
| S06090 | Concussion with loss of consciousness of unspecified duration without open intracranial wound | 0.6 (0.53, 0.67) | 0.57 (0.48, 0.68) |
| V184 | Pedal cyclist injured in noncollision transport accident, driver, traffic accident | 0.6 (0.52, 0.69) | 0.55 (0.45, 0.68) |
| S0190 | Open wound of head, part unspecified, uncomplicated | 0.56 (0.45, 0.71) | 0.55 (0.4, 0.77) |
| S0685 | Other intracranial injuries without open intracranial wound | 0.65 (0.59, 0.71) | 0.55 (0.48, 0.63) |
| E871 | Hypo-osmolality and hyponatraemia | 0.58 (0.47, 0.72) | 0.54 (0.4, 0.74) |
| S06010 | Concussion with brief loss of consciousness without open intracranial wound | 0.58 (0.54, 0.62) | 0.54 (0.49, 0.59) |
| N179 | Acute renal failure, unspecified | 0.4 (0.3, 0.53) | 0.53 (0.37, 0.77) |
| W5105 | Striking against or bumped into by another person in baseball | 0.54 (0.41, 0.71) | 0.52 (0.36, 0.77) |
| S22100 | Multiple fractures of thoracic spine, closed | 0.53 (0.41, 0.69) | 0.52 (0.35, 0.78) |
| T068 | Other specified injuries involving multiple body regions | 0.43 (0.36, 0.51) | 0.52 (0.41, 0.66) |
| S06500 | Traumatic subdural haemorrhage without loss of consciousness without open intracranial wound | 0.45 (0.39, 0.52) | 0.52 (0.43, 0.63) |
| E1152 | Type 2 diabetes mellitus with certain circulatory complications | 0.49 (0.38, 0.64) | 0.52 (0.37, 0.73) |
| S06810 | Other intracranial injuries with brief loss of consciousness without open intracranial wound | 0.37 (0.27, 0.51) | 0.51 (0.35, 0.74) |
| R410 | Disorientation, unspecified | 0.4 (0.31, 0.5) | 0.51 (0.37, 0.7) |
| S06300 | Focal brain injury without loss of consciousness, without open intracranial wound | 0.36 (0.27, 0.49) | 0.51 (0.34, 0.75) |
| S066 | Traumatic subarachnoid haemorrhage | 0.56 (0.53, 0.6) | 0.51 (0.46, 0.56) |
| S27100 | Traumatic haemothorax, without open wound into thoracic cavity | 0.56 (0.42, 0.74) | 0.5 (0.34, 0.74) |
| S0686 | Other intracranial injuries with open intracranial wound | 0.4 (0.27, 0.57) | 0.5 (0.31, 0.81) |
| S22490 | Multiple fractures of unspecified number of ribs, closed | 0.36 (0.29, 0.45) | 0.49 (0.37, 0.67) |
| S0100 | Open wound of scalp, uncomplicated | 0.48 (0.45, 0.52) | 0.49 (0.44, 0.55) |
| Z751 | Person awaiting admission to adequate facility elsewhere | 0.48 (0.43, 0.53) | 0.49 (0.42, 0.56) |
| U990 | While engaged in sports | 0.47 (0.39, 0.57) | 0.49 (0.37, 0.63) |
| R413 | Other amnesia | 0.57 (0.44, 0.75) | 0.49 (0.33, 0.71) |
| S27200 | Traumatic haemopneumothorax, without open wound into thoracic cavity | 0.41 (0.34, 0.5) | 0.48 (0.38, 0.61) |
| S0120 | Open wound of nose, uncomplicated | 0.41 (0.32, 0.51) | 0.48 (0.34, 0.68) |
| Y838 | Other surgical procedures as the cause of abnormal reaction or later complication, without mention of misadventure at the time of the procedure | 0.51 (0.42, 0.62) | 0.48 (0.36, 0.64) |
| R568 | Other and unspecified convulsions | 0.42 (0.33, 0.52) | 0.48 (0.35, 0.65) |
| S058 | Other injuries of eye and orbit | 0.36 (0.26, 0.51) | 0.48 (0.31, 0.74) |
| S42090 | Fracture of unspecified part of clavicle, closed | 0.46 (0.38, 0.56) | 0.47 (0.36, 0.62) |
| S32000 | Fracture of lumbar vertebra, L1 level, closed | 0.41 (0.31, 0.54) | 0.47 (0.33, 0.67) |
| S27000 | Traumatic pneumothorax, without open wound into thoracic cavity | 0.45 (0.4, 0.52) | 0.47 (0.39, 0.56) |
| S06640 | Traumatic subarachnoid haemorrhage with prolonged loss of consciousness without return to pre-existing level of consciousness without open intracranial wound | 0.53 (0.37, 0.76) | 0.46 (0.27, 0.79) |
| S065 | Traumatic subdural haemorrhage | 0.47 (0.44, 0.5) | 0.46 (0.42, 0.51) |
| S5100 | Open wound of elbow, uncomplicated | 0.42 (0.3, 0.59) | 0.46 (0.29, 0.73) |
| S12200 | Fracture of C3 - C4 vertebra, closed | 0.38 (0.26, 0.56) | 0.46 (0.28, 0.74) |
| Z235 | Need for immunization against tetanus alone | 0.5 (0.36, 0.7) | 0.46 (0.28, 0.74) |
| S42010 | Fracture of shaft of clavicle, closed | 0.51 (0.42, 0.63) | 0.45 (0.33, 0.63) |
| V180 | Pedal cyclist injured in noncollision transport accident, driver, nontraffic accident | 0.53 (0.48, 0.59) | 0.45 (0.39, 0.53) |
| S32010 | Fracture of lumbar vertebra, L2 level, closed | 0.28 (0.2, 0.39) | 0.45 (0.29, 0.7) |
| S06990 | Intracranial injury, unspecified with loss of consciousness of unspecified duration without open intracranial wound | 0.57 (0.43, 0.75) | 0.45 (0.3, 0.67) |
| S22410 | Multiple fractures of 5 or more ribs, closed | 0.4 (0.33, 0.47) | 0.45 (0.35, 0.57) |
| R5688 | Other and unspecified convulsions | 0.43 (0.36, 0.53) | 0.45 (0.34, 0.58) |
| S06690 | Traumatic subarachnoid haemorrhage with loss of consciousness of unspecified duration without open intracranial wound | 0.39 (0.31, 0.48) | 0.45 (0.33, 0.6) |
| G9381 | Neurologically determined death | 0.39 (0.27, 0.57) | 0.44 (0.27, 0.72) |
| S06210 | Diffuse brain injury with brief loss of consciousness without open intracranial wound | 0.46 (0.35, 0.62) | 0.44 (0.29, 0.68) |
| S12210 | Fracture of C5 - C7 vertebra, closed | 0.39 (0.33, 0.46) | 0.44 (0.34, 0.57) |
| X59 | Exposure to unspecified factor | 0.39 (0.31, 0.48) | 0.44 (0.33, 0.59) |
| F059 | Delirium, unspecified | 0.31 (0.22, 0.43) | 0.44 (0.29, 0.67) |
| S22200 | Fracture of sternum, closed | 0.48 (0.37, 0.61) | 0.44 (0.31, 0.63) |
| J90 | Pleural effusion, not elsewhere classified | 0.51 (0.37, 0.71) | 0.44 (0.27, 0.72) |
| S22300 | Fracture of rib, closed | 0.57 (0.48, 0.67) | 0.44 (0.35, 0.55) |
| F141 | Mental and behavioural disorders due to use of cocaine, harmful use | 0.41 (0.27, 0.62) | 0.44 (0.25, 0.76) |
| S22500 | Flail chest, closed | 0.4 (0.3, 0.52) | 0.43 (0.3, 0.63) |
| Z720 | Tobacco use | 0.4 (0.29, 0.55) | 0.43 (0.28, 0.68) |
| S0180 | Open wounds of other parts of head, uncomplicated | 0.44 (0.41, 0.47) | 0.43 (0.39, 0.48) |
| S06390 | Focal brain injury with loss of consciousness of unspecified duration without open intracranial wound | 0.29 (0.19, 0.43) | 0.43 (0.26, 0.71) |
| S06890 | Other intracranial injuries with loss of consciousness of unspecified duration without open intracranial wound | 0.34 (0.25, 0.48) | 0.43 (0.28, 0.66) |
| S0625 | Diffuse brain injury without open intracranial wound | 0.46 (0.42, 0.51) | 0.43 (0.37, 0.5) |
| S059 | Injury of eye and orbit, unspecified | 0.3 (0.22, 0.41) | 0.43 (0.3, 0.63) |
| S62390 | Fracture of unspecified site of other metacarpal bone, closed | 0.52 (0.34, 0.81) | 0.43 (0.24, 0.77) |
| S06200 | Diffuse brain injury without loss of consciousness without open intracranial wound | 0.4 (0.32, 0.5) | 0.42 (0.31, 0.58) |
| S36800 | Haematoma of other intra-abdominal organs without open wound into cavity | 0.54 (0.39, 0.73) | 0.42 (0.28, 0.64) |
| F103 | Mental and behavioural disorders due to use of alcohol, withdrawal state | 0.28 (0.22, 0.36) | 0.42 (0.31, 0.56) |
| S27300 | Contusion and haematoma of lung without open wound into thoracic cavity | 0.41 (0.36, 0.46) | 0.42 (0.35, 0.5) |
| S025 | Fracture of tooth | 0.44 (0.36, 0.53) | 0.42 (0.32, 0.55) |
| S22400 | Multiple fractures of 2 - 4 ribs, closed | 0.42 (0.37, 0.48) | 0.42 (0.34, 0.51) |
| Y08 | Assault by other specified means | 0.45 (0.33, 0.61) | 0.42 (0.28, 0.62) |
| S06910 | Intracranial injury, unspecified with brief loss of consciousness without open intracranial wound | 0.44 (0.36, 0.54) | 0.42 (0.31, 0.56) |
| S06290 | Diffuse brain injury with loss of consciousness of unspecified duration without open intracranial wound | 0.27 (0.21, 0.36) | 0.41 (0.28, 0.61) |
| S6100 | Open wound of finger(s) without damage to nail, uncomplicated | 0.41 (0.29, 0.57) | 0.41 (0.25, 0.68) |
| S02900 | Fracture of skull and facial bones, part unspecified, closed | 0.43 (0.38, 0.48) | 0.41 (0.35, 0.48) |
| Y919 | Alcohol involvement, not otherwise specified | 0.56 (0.48, 0.64) | 0.41 (0.33, 0.51) |
| S02101 | Fracture of base of skull, open | 0.27 (0.2, 0.37) | 0.41 (0.27, 0.61) |
| S061 | Traumatic cerebral oedema | 0.52 (0.39, 0.69) | 0.4 (0.27, 0.6) |
| S02800 | Fracture of alveolus, closed | 0.45 (0.36, 0.56) | 0.4 (0.29, 0.56) |
| S12700 | Multiple fractures of cervical spine, closed | 0.29 (0.2, 0.41) | 0.4 (0.26, 0.61) |
| S02400 | Fracture of malar and maxillary bones, LeFort 1, closed | 0.27 (0.2, 0.37) | 0.4 (0.26, 0.62) |
| Z590 | Homelessness | 0.52 (0.38, 0.7) | 0.4 (0.25, 0.63) |
| S02100 | Fracture of base of skull, closed | 0.37 (0.34, 0.39) | 0.4 (0.36, 0.44) |
| T797 | Traumatic subcutaneous emphysema | 0.29 (0.21, 0.39) | 0.4 (0.27, 0.59) |
| Y831 | Surgical operation with implant of artificial internal device as the cause of abnormal reaction or later complication, without mention of misadventure at the time of the procedure | 0.49 (0.41, 0.6) | 0.39 (0.3, 0.52) |
| V174 | Pedal cyclist injured in collision with fixed or stationary object, driver, traffic accident | 0.33 (0.21, 0.51) | 0.39 (0.21, 0.73) |
| S06590 | Traumatic subdural haemorrhage with loss of consciousness of unspecified duration without open intracranial wound | 0.37 (0.3, 0.46) | 0.39 (0.29, 0.53) |
| S82200 | Fracture of shaft of tibia with or without fibula, closed | 0.55 (0.41, 0.74) | 0.39 (0.25, 0.61) |
| S06610 | Traumatic subarachnoid haemorrhage with brief loss of consciousness without intracranial wound | 0.56 (0.44, 0.7) | 0.39 (0.27, 0.56) |
| J9810 | Atelectasis | 0.46 (0.33, 0.66) | 0.39 (0.22, 0.69) |
| W31 | Contact with other and unspecified machinery | 0.4 (0.3, 0.52) | 0.38 (0.26, 0.57) |
| S72300 | Fracture of shaft of femur, closed | 0.49 (0.37, 0.66) | 0.38 (0.26, 0.56) |
| S0181 | Open wounds of other parts of head, complicated | 0.28 (0.17, 0.47) | 0.38 (0.2, 0.72) |
| S02891 | Fracture of other and unspecified skull and facial bones NEC, open | 0.18 (0.1, 0.33) | 0.38 (0.19, 0.74) |
| W0203 | Fall involving skateboard | 0.32 (0.26, 0.39) | 0.38 (0.29, 0.5) |
| T814 | Infection following a procedure, not elsewhere classified | 0.43 (0.33, 0.56) | 0.37 (0.25, 0.56) |
| S6190 | Open wound of wrist and hand part, part unspecified, uncomplicated | 0.48 (0.33, 0.7) | 0.37 (0.21, 0.65) |
| J189 | Pneumonia, unspecified | 0.37 (0.3, 0.45) | 0.37 (0.28, 0.5) |
| S22000 | Fracture of thoracic vertebra T1 - T6, closed | 0.45 (0.37, 0.54) | 0.37 (0.28, 0.49) |
| S06510 | Traumatic subdural haemorrhage with brief loss of consciousness without open intracranial wound | 0.42 (0.33, 0.54) | 0.37 (0.26, 0.52) |
| Y848 | Other medical procedures as the cause of abnormal reaction or later complication, without mention of misadventure at the time of the procedure | 0.39 (0.33, 0.47) | 0.37 (0.28, 0.47) |
| S02430 | Fracture of malar and maxillary bones, LeFort 3, unilateral, closed | 0.3 (0.19, 0.48) | 0.36 (0.2, 0.68) |
| S0626 | Diffuse brain injury with open intracranial wound | 0.2 (0.12, 0.33) | 0.36 (0.19, 0.68) |
| S064 | Epidural haemorrhage | 0.34 (0.29, 0.4) | 0.36 (0.29, 0.46) |
| F100 | Mental and behavioural disorders due to use of alcohol, acute intoxication | 0.36 (0.32, 0.4) | 0.36 (0.31, 0.42) |
| V134 | Pedal cyclist injured in collision with car, pick-up truck or van, driver, traffic accident | 0.44 (0.37, 0.52) | 0.36 (0.29, 0.46) |
| S82201 | Fracture of shaft of tibia with or without fibula, open | 0.42 (0.29, 0.59) | 0.36 (0.2, 0.62) |
| S0635 | Focal brain injury without open intracranial wound | 0.38 (0.34, 0.44) | 0.35 (0.29, 0.43) |
| S02810 | Fracture of hard palate, closed | 0.35 (0.21, 0.57) | 0.35 (0.17, 0.71) |
| W24 | Contact with lifting and transmission devices, not elsewhere classified | 0.2 (0.11, 0.38) | 0.35 (0.17, 0.71) |
| S097 | Multiple injuries of head | 0.22 (0.15, 0.32) | 0.35 (0.22, 0.57) |
| B965 | Pseudomonas (aeruginosa) as the cause of diseases classified to other chapters | 0.39 (0.25, 0.63) | 0.35 (0.18, 0.68) |
| W27 | Contact with nonpowered hand tool | 0.17 (0.1, 0.28) | 0.35 (0.19, 0.62) |
| S02201 | Fracture of nasal bones, open | 0.27 (0.16, 0.44) | 0.34 (0.18, 0.65) |
| B956 | Staphylococcus aureus as the cause of diseases classified to other chapters | 0.24 (0.17, 0.34) | 0.34 (0.22, 0.54) |
| S22010 | Fracture of thoracic vertebra T7- T12, closed | 0.33 (0.26, 0.42) | 0.34 (0.24, 0.47) |
| I4890 | Atrial fibrillation, unspecified | 0.49 (0.32, 0.76) | 0.34 (0.17, 0.66) |
| S0150 | Open wound of lip and oral cavity, uncomplicated | 0.38 (0.32, 0.44) | 0.34 (0.27, 0.42) |
| F058 | Other delirium | 0.27 (0.18, 0.42) | 0.34 (0.18, 0.64) |
| S02200 | Fracture of nasal bones, closed | 0.32 (0.3, 0.35) | 0.34 (0.3, 0.38) |
| S06011 | Concussion with brief loss of consciousness with open intracranial wound | 0.47 (0.28, 0.77) | 0.33 (0.17, 0.64) |
| J9588 | Other postprocedural respiratory disorders | 0.33 (0.27, 0.41) | 0.33 (0.24, 0.45) |
| S0110 | Open wound of eyelid and periocular area, uncomplicated | 0.32 (0.27, 0.37) | 0.33 (0.27, 0.4) |
| S051 | Contusion of eyeball and orbital tissues | 0.37 (0.28, 0.48) | 0.33 (0.23, 0.48) |
| S02301 | Fracture of orbital floor, open | 0.23 (0.14, 0.4) | 0.33 (0.16, 0.65) |
| R33 | Retention of urine | 0.44 (0.28, 0.69) | 0.32 (0.16, 0.65) |
| G9388 | Other specified disorders of brain | 0.27 (0.19, 0.37) | 0.32 (0.2, 0.52) |
| V299 | Motorcycle rider [any] injured in unspecified traffic accident | 0.26 (0.16, 0.43) | 0.32 (0.17, 0.63) |
| F101 | Mental and behavioural disorders due to use of alcohol, harmful use | 0.37 (0.32, 0.44) | 0.32 (0.25, 0.41) |
| J9699 | Respiratory failure, unspecified, type unspecified | 0.24 (0.13, 0.46) | 0.32 (0.15, 0.69) |
| V139 | Pedal cyclist injured in collision with car, pick-up truck or van, unspecified pedal cyclist, traffic accident | 0.23 (0.11, 0.47) | 0.32 (0.15, 0.69) |
| S32020 | Fracture of lumbar vertebra, L3 level, closed | 0.4 (0.29, 0.56) | 0.32 (0.19, 0.53) |
| S050 | Injury of conjunctiva and corneal abrasion without mention of foreign body | 0.35 (0.25, 0.49) | 0.32 (0.19, 0.52) |
| S02000 | Fracture of vault of skull, closed | 0.28 (0.25, 0.32) | 0.32 (0.26, 0.38) |
| S06400 | Epidural haemorrhage without loss of consciousness without open intracranial wound | 0.27 (0.18, 0.4) | 0.31 (0.19, 0.51) |
| S02670 | Multiple mandibular fracture sites, closed | 0.35 (0.24, 0.51) | 0.31 (0.18, 0.54) |
| S06310 | Focal brain injury with brief loss of consciousness without open intracranial wound | 0.5 (0.34, 0.73) | 0.31 (0.18, 0.54) |
| S02890 | Fracture of other and unspecified skull and facial bones NEC, closed | 0.3 (0.28, 0.33) | 0.31 (0.28, 0.35) |
| H113 | Conjunctival haemorrhage | 0.38 (0.27, 0.52) | 0.31 (0.19, 0.51) |
| J152 | Pneumonia due to Staphylococcus | 0.26 (0.18, 0.37) | 0.31 (0.19, 0.5) |
| V8658 | Driver of other all-terrain or other off road motor vehicle injured in nontraffic accident | 0.29 (0.25, 0.34) | 0.31 (0.25, 0.39) |
| Y04 | Assault by bodily force | 0.31 (0.3, 0.33) | 0.31 (0.29, 0.33) |
| H532 | Diplopia | 0.62 (0.44, 0.86) | 0.31 (0.18, 0.52) |
| S02001 | Fracture of vault of skull, open | 0.27 (0.19, 0.37) | 0.31 (0.19, 0.5) |
| T796 | Traumatic ischaemia of muscle | 0.39 (0.26, 0.57) | 0.3 (0.17, 0.56) |
| S02480 | Other fracture of malar and maxillary bones, closed | 0.26 (0.23, 0.29) | 0.3 (0.25, 0.35) |
| F102 | Mental and behavioural disorders due to use of alcohol, dependence syndrome | 0.39 (0.32, 0.47) | 0.3 (0.22, 0.41) |
| S02300 | Fracture of orbital floor, closed | 0.3 (0.28, 0.32) | 0.3 (0.27, 0.32) |
| S02490 | Unspecified fracture of malar and maxillary bones, closed | 0.26 (0.22, 0.3) | 0.29 (0.24, 0.36) |
| R470 | Dysphasia and aphasia | 0.45 (0.3, 0.68) | 0.29 (0.15, 0.56) |
| J690 | Pneumonitis due to food and vomit | 0.32 (0.25, 0.41) | 0.28 (0.19, 0.42) |
| J14 | Pneumonia due to Haemophilus influenzae | 0.25 (0.16, 0.41) | 0.28 (0.13, 0.61) |
| S42180 | Fracture of other part of scapula, closed | 0.29 (0.2, 0.41) | 0.28 (0.16, 0.5) |
| F104 | Mental and behavioural disorders due to use of alcohol, withdrawal state with delirium | 0.23 (0.15, 0.36) | 0.28 (0.15, 0.5) |
| W2202 | Striking against or struck by/playing hockey | 0.3 (0.26, 0.34) | 0.27 (0.23, 0.33) |
| J80 | Adult respiratory distress syndrome | 0.36 (0.23, 0.57) | 0.27 (0.15, 0.51) |
| S02600 | Fracture of mandible, closed | 0.33 (0.27, 0.41) | 0.27 (0.19, 0.38) |
| V193 | Pedal cyclist [any] injured in unspecified nontraffic accident | 0.3 (0.18, 0.49) | 0.27 (0.12, 0.61) |
| S43100 | Dislocation of acromioclavicular joint, closed | 0.31 (0.22, 0.44) | 0.27 (0.17, 0.43) |
| S02700 | Multiple fractures involving skull and facial bones, closed | 0.25 (0.22, 0.29) | 0.27 (0.22, 0.32) |
| Z722 | Drug use | 0.32 (0.21, 0.5) | 0.26 (0.13, 0.54) |
| W5102 | Striking against or bumped into by another person in hockey | 0.28 (0.26, 0.31) | 0.26 (0.22, 0.3) |
| S42190 | Fracture of unspecified part of scapula, closed | 0.23 (0.17, 0.31) | 0.26 (0.17, 0.39) |
| V274 | Motorcycle rider injured in collision with fixed or stationary object, driver, traffic accident | 0.12 (0.06, 0.24) | 0.26 (0.13, 0.53) |
| S82301 | Fracture of lower (distal) end of tibia with or without fibula, open | 0.41 (0.24, 0.7) | 0.25 (0.11, 0.6) |
| W2102 | Striking against or struck by hockey stick | 0.26 (0.2, 0.36) | 0.25 (0.17, 0.37) |
| U986 | Place of occurrence, industrial and construction area | 0.23 (0.21, 0.26) | 0.25 (0.21, 0.29) |
| S0130 | Open wound of ear, uncomplicated | 0.27 (0.21, 0.36) | 0.25 (0.17, 0.36) |
| U821 | Resistance to methicillin | 0.33 (0.19, 0.59) | 0.25 (0.09, 0.64) |
| Y839 | Surgical procedure, unspecified, as the cause of abnormal reaction or later complication, without mention of misadventure at the time of the procedure | 0.47 (0.32, 0.71) | 0.25 (0.11, 0.56) |
| W80 | Inhalation and ingestion of other objects causing obstruction of respiratory tract | 0.21 (0.12, 0.39) | 0.25 (0.12, 0.53) |
| I2510 | Atherosclerotic heart disease of native coronary artery | 0.2 (0.11, 0.36) | 0.24 (0.12, 0.48) |
| V8650 | Driver of snowmobile injured in nontraffic land accident | 0.34 (0.26, 0.44) | 0.24 (0.16, 0.36) |
| S02440 | Fracture of malar and maxillary bones, LeFort 3, bilateral, closed | 0.26 (0.16, 0.42) | 0.24 (0.12, 0.49) |
| R451 | Restlessness and agitation | 0.21 (0.15, 0.29) | 0.24 (0.16, 0.37) |
| S02901 | Fracture of skull and facial bones, part unspecified, open | 0.35 (0.2, 0.6) | 0.24 (0.11, 0.54) |
| U99011 | Ice hockey | 0.3 (0.26, 0.35) | 0.24 (0.19, 0.3) |
| T179 | Foreign body in respiratory tract, part unspecified | 0.31 (0.14, 0.68) | 0.24 (0.09, 0.62) |
| B182 | Chronic viral hepatitis C | 0.27 (0.16, 0.47) | 0.24 (0.12, 0.48) |
| W2103 | Striking against or struck by hockey puck | 0.24 (0.19, 0.3) | 0.24 (0.17, 0.32) |
| S02420 | Fracture of malar and maxillary bones, combined LeFort 1 with LeFort 2 (contralateral fractures), closed | 0.21 (0.16, 0.29) | 0.23 (0.15, 0.37) |
| S004 | Superficial injury of ear | 0.23 (0.13, 0.39) | 0.23 (0.11, 0.5) |
| A419 | Sepsis, unspecified | 0.31 (0.2, 0.47) | 0.23 (0.12, 0.44) |
| Y09 | Assault by unspecified means | 0.21 (0.17, 0.25) | 0.23 (0.18, 0.29) |
| W11 | Fall on and from ladder | 0.3 (0.26, 0.33) | 0.23 (0.19, 0.28) |
| R001 | Bradycardia, unspecified | 0.34 (0.21, 0.56) | 0.22 (0.1, 0.49) |
| X99 | Assault by sharp object | 0.2 (0.15, 0.28) | 0.22 (0.13, 0.35) |
| V585 | Occupant of pick-up truck or van injured in noncollision transport accident, driver, traffic accident | 0.32 (0.22, 0.47) | 0.22 (0.11, 0.41) |
| S06410 | Epidural haemorrhage with brief loss of consciousness without open intracranial wound | 0.25 (0.14, 0.43) | 0.21 (0.1, 0.45) |
| S250 | Injury of thoracic aorta | 0.33 (0.18, 0.61) | 0.21 (0.09, 0.5) |
| F209 | Schizophrenia, unspecified | 0.32 (0.21, 0.5) | 0.21 (0.1, 0.42) |
| I480 | Atrial fibrillation | 0.37 (0.23, 0.6) | 0.21 (0.11, 0.41) |
| W13 | Fall from, out of or through building or structure | 0.23 (0.19, 0.28) | 0.21 (0.15, 0.28) |
| S0170 | Multiple open wounds of head, uncomplicated | 0.29 (0.23, 0.38) | 0.2 (0.13, 0.31) |
| V284 | Motorcycle rider injured in noncollision transport accident, driver, traffic accident | 0.29 (0.22, 0.38) | 0.2 (0.13, 0.3) |
| Y00 | Assault by blunt object | 0.2 (0.18, 0.24) | 0.2 (0.16, 0.25) |
| X590 | Exposure to unspecified factor causing fracture | 0.3 (0.22, 0.4) | 0.19 (0.12, 0.3) |
| S42120 | Fracture of glenoid cavity and neck of scapula, closed | 0.22 (0.11, 0.43) | 0.19 (0.07, 0.54) |
| Y356 | Legal intervention involving other specified means | 0.22 (0.13, 0.35) | 0.19 (0.09, 0.4) |
| S02610 | Fracture of ramus, closed | 0.17 (0.09, 0.32) | 0.18 (0.08, 0.41) |
| Y901 | Blood alcohol level of 20-39 mg/100 ml | 0.27 (0.13, 0.55) | 0.18 (0.06, 0.52) |
| V293 | Motorcycle rider [any] injured in unspecified nontraffic accident | 0.15 (0.05, 0.49) | 0.18 (0.05, 0.59) |
| S0140 | Open wound of cheek and temporomandibular area, uncomplicated | 0.23 (0.17, 0.33) | 0.17 (0.1, 0.3) |
| R780 | Finding of alcohol in blood | 0.3 (0.18, 0.5) | 0.16 (0.06, 0.41) |
| S02701 | Multiple fractures involving skull and facial bones, open | 0.33 (0.2, 0.53) | 0.16 (0.06, 0.41) |
| V280 | Motorcycle rider injured in noncollision transport accident, driver, nontraffic accident | 0.15 (0.1, 0.22) | 0.14 (0.08, 0.25) |
| V8608 | Driver of other all-terrain or other off road motor vehicle injured in traffic accident | 0.12 (0.06, 0.23) | 0.14 (0.06, 0.35) |
| S37090 | Injury of kidney NOS without open wound into cavity | 0.41 (0.23, 0.72) | 0.13 (0.05, 0.36) |
| V234 | Motorcycle rider injured in collision with car, pick-up truck or van, driver, traffic accident | 0.15 (0.11, 0.2) | 0.12 (0.08, 0.2) |
| U99003 | Football | 0.18 (0.13, 0.26) | 0.12 (0.06, 0.21) |
| Y902 | Blood alcohol level of 40-59 mg/100 ml | 0.33 (0.19, 0.59) | 0.11 (0.03, 0.37) |
| S02410 | Fracture of malar and maxillary bones, LeFort 2, closed | 0.15 (0.09, 0.25) | 0.1 (0.04, 0.25) |
| R318 | Other and unspecified hematuria | 0.3 (0.15, 0.58) | 0.08 (0.02, 0.33) |
| W12 | Fall on and from scaffolding | 0.04 (0.02, 0.1) | 0.08 (0.03, 0.22) |
| Z21 | Asymptomatic human immunodeficiency virus [HIV] infection status | 0.14 (0.06, 0.33) | 0.04 (0.01, 0.28) |
| V685 | Occupant of heavy transport vehicle injured in noncollision transport accident, driver, traffic accident | 0.05 (0.01, 0.19) | 0.04 (0.01, 0.27) |

**Supplementary Table 6. Sex and gender score effect sizes (OR and 95% CI) for predicting discharge location subcategories of “Other” vs. “Home” (test set, N = 4,427).** Logistic regression models controlled for *age, LOS, ADG score, rurality*, and *income quintile.* Models A-D based on records prior to 2018 (at later dates discharge locations in these categories were not used).

Abbreviations: OR = odds ratio, CI = confidence interval; LAMA = Left Against Medical Advice.

*Number of subjects within respective subcategory of “Other” discharge location

| **Model** | **n*** | ***Gender score*** | ***Sex*** |
| --- | --- | --- | --- |
|  |  | **OR (95% CI)** | **OR (95% CI)** |
| Model A: Another hospital/acute care facility | 676 | 0.22 (0.13, 0.40) | 1.23 (1.00, 1.52)) |
| Model B: Long term/continued | 43 | 0.92 (0.14, 6.14) | 0.62 (0.28, 1.40) |
| Model C: Other ambulatory care/palliative care/hospice, addiction treatment, jails | 77 | 0.58 (0.13, 2.53) | 0.39 (0.19, 0.78) |
| Model D. Signed out against medical advice | 153 | 0.63 (0.22, 1.77) | 0.48 (0.30, 0.76) |
| Model E. Inpatient care | 118 | 0.07 (0.02, 0.26) | 1.31 (0.84, 2.04) |
| Model F. Died in facility | 72 | 0.35 (0.08, 1.58) | 0.96 (0.54, 1.69) |
| Model G: LAMA | 24 | 0.40 (0.03, 5.35) | 0.73 (0.25, 2.07) |
